# Evidence-Based ZHENG: A Traditional Chinese Medicine Syndrome 2013

**DOI:** 10.1155/2014/484201

**Published:** 2014-05-06

**Authors:** Shi-Bing Su, Wei Jia, Aiping Lu, Shao Li

**Affiliations:** ^1^Research Center for Complex System of Traditional Chinese Medicine, Shanghai University of Traditional Chinese Medicine, Shanghai 201203, China; ^2^University of Hawaii Cancer Center, Honolulu, HI 96813, USA; ^3^School of Chinese Medicine, Hong Kong Baptist University, Kowloon Tong, Hong Kong; ^4^Bioinformatics Division, TNLIST and Department of Automation, Tsinghua University, Beijing 100084, China

ZHENG, also known as traditional Chinese medicine (TCM) syndrome or TCM pattern, is an integral and essential part of TCM theory. A TCM ZHENG, in essence, is a characteristic profile of all clinical manifestations that can be identified by a TCM practitioner. Clinical treatments of a patient rely on the successful differentiation of a specific ZHENG. Recently, technologies and methods of omics, bioinformatics, bionetwork, and data mining through a system biology approach were applied in ZHENG research, which have significantly facilitated the development of ZHENG theory. Following 2012 Evidence-Based ZHENG special issue, we have further grouped together 15 excellent papers on TCM ZHENG research and put them forward for publication in this special issue.

Firstly, there are 2 research papers in this special issue which addressed the clinical trials based on TCM ZHENG classification and treatment. A paper has evaluated the adaptability of TCM ZHENG diagnosis and found that “Yang-Xu Zheng” of TCM is an early prognostic predictor for patients with severe sepsis and septic shock. Another paper, with a randomized, double-blind clinical study, has evaluated the efficacy of crest herbal toothpaste in “clearing internal heat.” Interestingly it has been instructed that the toothpaste (Crest Herbal Crystal) is efficacious through brushing with 1 g toothpaste for 2 minutes each time, 2 times per day in 4 weeks in the internal heat. In addition, the studies of the acupuncture, an efficient therapeutic method originated in ancient China, have also been systematically reviewed.

In TCM, the clinical diagnosis of ZHENG relies on the gathering of clinical information through inspection, auscultation and olfaction, inquiry, and palpation. For ZHENG measurement of the inquiry, 2 research articles of this special issue presented the ZHENG diagnostic methods. A research article discussed the method of objective auscultation of TCM based on wavelet packet fractal dimension and support vector machine. Another paper has investigated ZHENG classification with missing feature values using local-validity approach. This is through the study of the value contributed by symptoms to syndrome diagnosis of chronic hepatitis B, that is, diagnosis weight, constructing symptom-syndrome scale of chronic hepatitis B, and realizing quantitative diagnosis from symptom to syndrome.

Five research articles of this special issue presented the TCM ZHENG classification using profiles of genes, proteins, metabolites, and/or biomarkers. Therein, it has been identified as the relationship between EGF, TGFA, and EGFR gene polymorphisms and ZHENG in gastric cancer, and the curative effects of ZHENG-based Fuzheng-Huayu tablet on hepatitis B caused cirrhosis related to CYP1A2 genetic polymorphism. Moreover, a study of rheumatoid arthritis with deficiency pattern has showed the correlation with cold and hot patterns in gene expression profiles. A new biomarker feature pattern has been identified consisting of TNF-a, IL-10, and IL-8 for blood stasis syndrome corresponding to myocardial ischemia. Additionally, recent advances in metabolomics study of TCM ZHENG have been reviewed.

Bioinformatics and bionetwork are becoming a cutting-edge research field in current TCM ZHENG and ZHENG-based treatment studies. In this special issue, a research article analyzed the characteristics of ZHENG from excessive to deficient syndromes in hepatocarcinoma underlying miRNA array data using bioinformatics and bionetwork techniques. Moreover, a network-based systematic study presented the mechanism of ZHENG related treatment in patients with cough variant asthma, suggesting that the network-based systematic study will be a good way to improve the scientific understanding of mechanism of the treatment of ZHENG.

In TCM, ZHENG experimental models are necessary tools for the mechanistic study of ZHENG and the evaluation of TCM treatment. In this special issue, a review paper summarized the utilization of model organisms in the construction of TCM ZHENG experimental model and highlighted the relevance of modern medicine with ZHENG animal model. Moreover, a paper reported the establishment of an experimental breast cancer ZHENG model and the ZHENG-based curative effect evaluation of a Chinese medicine formula Zuo-Jin Wan. Furthermore, the protective effects of two herbal extract and their metabolic signatures in rat kidney yang deficiency syndrome model have been also presented. More reports on ZHENG experimental models are expected in the near future.

In summary, the concept of TCM ZHENG, as a main diagnostic approach in TCM, would provide invaluable guidance for the therapeutic choices and personalized disease management, not only in traditional medical practices but in modern healthcare systems as well. We look forward to an increasing number and sizes of clinical trials and experimental approaches utilizing TCM ZHENG to be conducted in the future to further promote the development of evidence-based personalized medicine.

*Shi-Bing  Su* *Shi-Bing  Su* *Wei Jia* *Wei Jia* *Aiping  Lu* *Aiping  Lu* *Shao Li* *Shao Li*

